# A method to analyze the influence of mechanical strain on dermal collagen morphologies

**DOI:** 10.1038/s41598-021-86907-7

**Published:** 2021-04-07

**Authors:** Maximilian Witte, Michael Rübhausen, Sören Jaspers, Horst Wenck, Frank Fischer

**Affiliations:** 1 Center for Free-Electron Laser Science (CFEL), University of Hamburg, Hamburg, 22607 Germany; 2 Beiersdorf AG, Hamburg, 20245 Germany

**Keywords:** Computational biophysics, Tissues, Mechanical engineering

## Abstract

Collagen fibers and their orientation play a major role in the mechanical behavior of soft biological tissue such as skin. Here, we present a proof-of-principle study correlating mechanical properties with collagen fiber network morphologies. A dedicated multiphoton stretching device allows for mechanical deformations in combination with a simultaneous analysis of its collagen fiber network by second harmonic generation imaging (SHG). The recently introduced Fiber Image Network Evaluation (FINE) algorithm is used to obtain detailed information about the morphology with regard to fiber families in collagen network images. To demonstrate the potential of our method, we investigate an isotropic and an anisotropic ex-vivo dorsal pig skin sample under quasi-static cyclic stretching and relaxation sequences. Families of collagen fibers are found to form a partially aligned collagen network under strain. We find that the relative force uptake is accomplished in two steps. Firstly, fibers align within their fiber families and, secondly, fiber families orient in the direction of force. The maximum alignment of the collagen fiber network is found to be determined by the largest strain. Isotropic and anisotropic samples reveal a different micro structural behavior under repeated deformation leading to a similar force uptake after two stretching cycles. Our method correlates mechanical properties with morphologies in collagen fiber networks.

## Introduction

Skin is the largest organ of the human body and it is responsible for the bodys protection towards external factors. In our daily life skin is able to undergo large strains of up to $25\,\%$^1^. The mechanical functionality of skin is altered due to aging^2^, scars^3^ and skin diseases like the Ehlers-Danlos syndrome^4^. The microstructural identification and quantification of these factors is crucial for the development of counteractive biological approaches.

Collagen fibers are known to dominate the mechanical properties of skin and are also one of its main constituents^5–8^. Upon stretching of skin, collagen fibers straighten, align in the direction of force and, at high strains, slide against each other^9,10^. There is a discussion about the onset of fiber alignment in the literature^6^. It is assumed that the unstressed orientation distribution of the dermal collagen fiber network defines the anisotropic mechanical behavior of skin^11,12^. Furthermore, diverse viscoelastic properties such as creep, stress relaxation, strain history-dependence, and strain-rate dependence are believed to depend on the collagen fiber network^13–18^. Indeed previous second harmonic generation (SHG) imaging^19^ at different stretching states has shown that the fibers orient along the force direction^6,10^. However, a microstructural interpretation of the observed changes of collagen networks requires a suitable approach to characterize and quantify complex fiber networks in fiber-reinforced materials.

In order to understand the properties of the dermal collagen fiber network under cyclic loads, we use a dedicated multiphoton stretching device and determine the network properties by the recently introduced Fiber Image Network Evaluation (FINE) algorithm^20,21^. The FINE algorithm evaluates the number of fiber families, their angular properties, and the alignment index. It is based on the cumulative orientation distribution^20^ and was successfully applied to a stack of in-vivo SHG images of human dermal collagen^21^. The FINE algorithm was also applied to silver-nanowire composites under mechanical strain^22^. Here, we present a method allowing for SHG imaging, while simultaneously deforming the sample. On each image representing a specific strain state, the FINE algorithm is applied to quantify the orientation of the collagen fiber network^21^.

## Results and discussion

### Sample stretching and image processing

**Figure 1:**
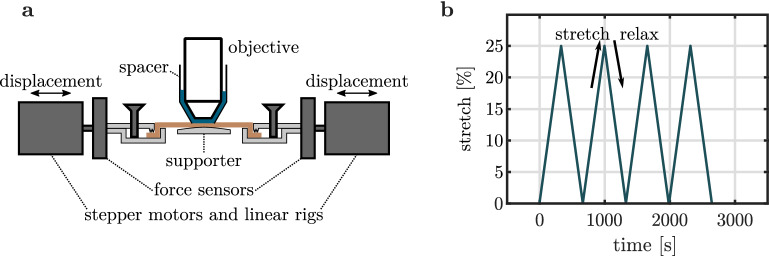
Experimental set-up and deformation protocol. (**a**) Schematic illustration of the multiphoton microscope stretching device. (**b**) Deformation protocol consisting of four repeated cycles with successive stretching and relaxation.

The multiphoton stretching device that is used for deforming skin, while simultaneously capturing its collagen fiber network, is shown in Fig. 1a and Supplementary Fig. 1. An exemplary deformation protocol is shown in Fig. 1b. Four consecutive cycles of repeated stretching and relaxation in cranial-caudal direction are applied to the skin. For our proof-of-principle study we use maximum strains of $25\,\%$ as it appears to be the maximum strain in the human body, which was measured at the forearm^1^. To ensure a reliable sequence of image acquisition and storage, images are recorded every $3\,\text {s}$ with a mean scan time of $(2.63\pm 0.04)\,\text {s}$. By using a rather low strain rate of $0.075\,\%\!/\!{\text {s}}$, compared to other studies^8,18^, the variation of strain during image acquisition is assumed to be negligible.

We use the FINE algorithm to obtain detailed, quantitative information about the collagen fiber network at each state of the deformation protocol^21^.

The FINE algorithm determines the number of fiber families *N* and their angular properties in terms of their mean orientations ${\bar{\theta }}_i$, their amplitudes $a_i$ and their dispersion parameters $b_i$. The dispersion parameter *b* describes the spread of fiber angles around their main orientation and increases with fiber alignment. To measure the overall alignment of the fiber network, we evaluate the alignment index (AI)^21^:

1 $$\begin{aligned} \text {AI}=\sum _1^{N}a_i\cdot b'_i\qquad b'_i = (b_i-b_\text {min})/(b_\text {max}-b_\text {min}), \end{aligned}$$

where $b_\text {min}=0.016$ and $b_\text {max}=0.26$ define the scale of the AI^21^. In addition, we make use of the orientation index (OI) in order to quantify the fraction of fibers that is oriented along the force direction of $90^\circ$:

2 $$\begin{aligned} \text {OI} = 2\frac{\sum _{\theta =0^\circ }^{180^\circ }{I}(\theta )\cos ^2(\theta -90^\circ )}{\sum _{\theta =0^\circ }^{180^\circ }{I}(\theta )}-1, \end{aligned}$$

where $I(\theta )$ denotes the angular orientation distribution, which is achieved by a Fourier-based method^20^. Note that the common definition of the OI uses the main orientation ${\bar{I}}(\theta )$ of the fiber network^23^.

Two rectangular skin samples originating from the same dorsal pig skin are analyzed. Samples were chosen such that they express a different unstressed orientation of their collagen fiber network as indicated by FINE parameters. In the process, an isotropic and an anisotropic sample were identified. An isotropic fiber network is indicated by low OI and AI values. This is the case for one of our samples having an OI of 0.05 and and AI of 0.18. That is the reason why we refer to this sample as *isotropic sample*. In contrast, the collagen fiber network of the *anisotropic sample* expresses an OI and an AI of 0.31 and 0.32, respectively. For more information about the FINE parameters and the detailed introduction of isotropic and anisotropic classes, we refer to our previous work^21^. The original, anatomical location of both samples is illustrated in Supplementary Fig. 2.

**Figure 2:**
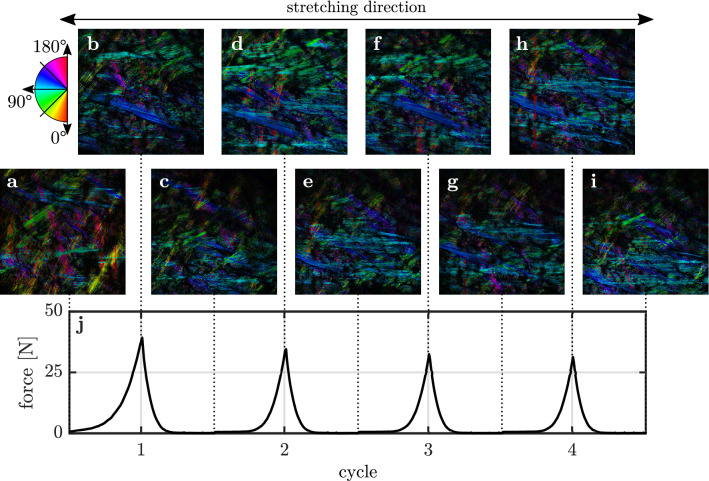
Evolution of the local collagen fiber orientation of the isotropic skin sample due to repeated stretching and relaxation. Local fiber orientations are shown in false colors. Dashed lines indicate the minimum and maximum strains of the corresponding stretching cycle. (**a**) Collagen fiber orientation prior to deformation. The sample is stretched in the $90^\circ$ direction as indicated by the arrow. (**b**) Local fiber orientation at maximum stretch of the first deformation cycle. The corresponding local orientations at maximum stretch of the second, third, and fourth deformation cycle are shown in (**d**), (**f**) and (**h**), respectively. (**c**) Local fiber orientation at maximum relaxation of the first deformation cycle. Similarly, local orientations at minimum stretch of the second, third, and fourth deformation cycle are shown in (**e**), (**g**) and (**i**), respectively. (**j**) Averaged force acting on the sample, measured by the force sensors as a function of the deformation cycle.

The collagen fiber network of the isotropic sample at minimum and maximum strain of the deformation protocol (Fig. 2b) is shown in Fig. 2(a–i). The measured averaged force acting onto the sample is shown in Fig. 2j. The corresponding figure for the anisotropic sample is shown in Supplementary Fig. 3. SHG images in Fig. 2a–i are processed to visualize the local orientation of collagen fibers in false colors.

As shown in Fig. 2a, the orientation of the collagen fibers initially occupies the entire angular range from $0^\circ$ to $180^\circ$. At maximum stretch of the first cycle, the fibers in Fig. 2b orient along the direction of force ($90^\circ$) with a variation of $\pm 45^\circ$. After complete relaxation of the sample, thick fiber bundles originally oriented along the $110^\circ$ direction have disappeared (Fig. 2c). Compared to the initial state in Fig. 2a, the fraction of fibers that are aligned along the force direction has strongly increased as visible by the enhanced amount of blue colors. At maximum stretch of the second deformation cycle (Fig. 2d), the fraction of oriented fibers is further increased compared to Fig. 2b. The fraction of oriented fibers further increases after the second deformation cycle in Fig. 2e, compared to Fig. 2c. This trend continues after the third and the fourth deformation cycle in Fig. 2g,i, respectively. Repeated stretching of the sample seems to have little effect on the local fiber orientation at maximum stretch, as seen in Fig. 2f,h.

### Collagen fiber network upon stretching

**Figure 3:**
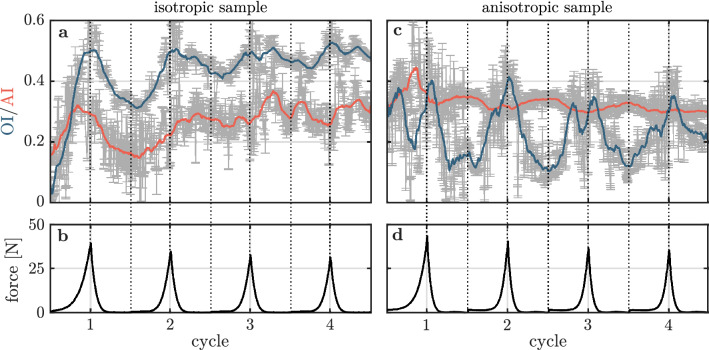
Derived parameters of the FINE algorithm applied to the collagen fiber network of the isotropic and the anisotropic sample under repeated stretching and relaxation. (**a**) Orientation index (OI) and alignment index (AI) of the collagen network of the isotropic sample as a function of the deformation cycle number. Data points with error bars indicating the $95\,\%$ confidence intervals are shown in grey. Solid lines represent smoothed data points. (**b**) Averaged force acting on the isotropic sample measured by the force sensors as a function of the deformation cycle number. (**c**) OI and AI of the collagen network of the anisotropic sample as a function of the deformation cycle number. (**d**) Averaged force acting on the anisotropic sample measured by the force sensors as a function of the deformation cycle number.

The OI and the AI of the isotropic sample are shown in Fig. 3a as a function of the deformation cycle number. The measured force is displayed in Fig. 3b. Initially, the isotropic sample is characterized by an OI of 0.05 and an AI of 0.16. This indicates an isotropic fiber network, where a negligible fraction of fibers is oriented along the direction of force. Upon stretching, both quantities increase until the OI reaches a maximum value of 0.50 at maximum sample stretch. This agrees with the local fiber orientation of Fig. 2b, where aligned fibers still express a considerable angular range. The AI reaches its highest value of 0.32 at $16\,\%$ of maximum stretch. Compared to their initial values, the OI and the AI are significantly increased after relaxation as they amount to 0.31 and 0.15, respectively. This trend continues throughout the additional deformation cycles until the OI and the AI fluctuate permanently around values of 0.48 and 0.30, respectively. This is visually in line with the local collagen fiber orientation of Fig. 2e–i, which hardly differ from each other after two deformation cycles. This suggests that collagen fibers of the isotropic sample are not relaxing to their initial orientation during the experiment.

The OI and the AI of the anisotropic sample are shown in Fig. 3c as a function of the deformation number. The corresponding measured force is displayed in Fig. 3d. The aligned network is characterized by an OI of 0.31 and an AI of 0.32 in the initial state. This OI additionally indicates that a non-vanishing fraction of fibers aligned along the direction of force before the initial stretching cycle. Stretching the anisotropic sample increases the AI to a local maximum of 0.44 at $18\,\%$ sample stretch. The OI first decreases, but then maximizes locally to 0.40 at maximum stretch. Similar to the isotropic sample, the collagen fibers first align themselves and then orient into the direction of stretch during the first deformation cycle. The observations of Bancelin *et al*., who measured a simultaneous increase of the OI with sample stretch, are verified^6^. The findings additionally suggest that repeated stretching and relaxation decreases the ability of the collagen network to orient along the direction of force. This is indicated by a continuously decreasing amplitude of the OI for both samples.

**Figure 4:**
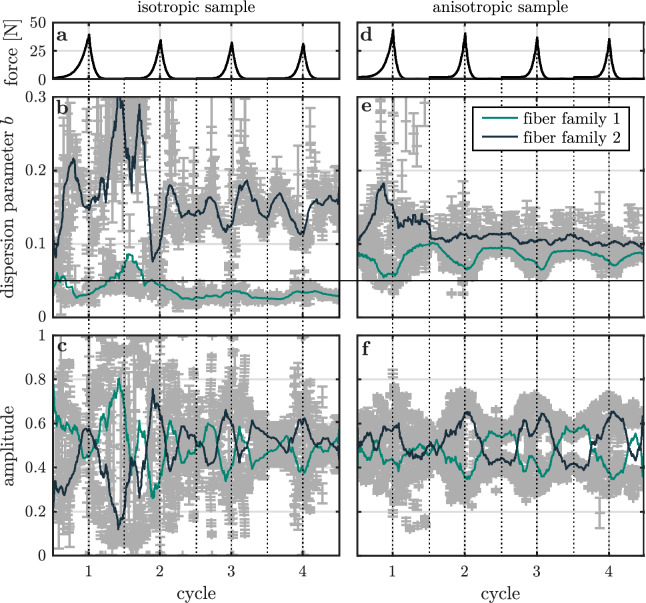
FINE algorithm parameters of the collagen fiber families of the isotropic and the anisotropic sample under repeated stretching and relaxation. (**a**) Averaged force acting on the isotropic sample, measured by the force sensors as a function of the deformation cycle number. (**b**) Dispersion parameter *b* of each fiber family identified by the FINE algorithm in the isotropic sample as a function of the deformation cycle number. Data points with error bars indicating the $95\,\%$ confidence intervals are shown in grey. Solid lines represent smoothed data points. Note that, a third fiber family is identified in a negligible fraction of images ($\le 1\,\%$). For the sake of clarity, parameters of this fiber family are not shown. The line at $b=0.05$ indicates the threshold value below which a fiber family is considered as isotropic. (**c**) Amplitude of each fiber family identified by the FINE algorithm in the isotropic sample as a function of the deformation cycle number. (**d**) Averaged force acting on the anisotropic sample, measured by the force sensors as a function of the deformation cycle number. (**e**) Dispersion parameter *b* of each fiber family identified by the FINE algorithm in the anisotropic sample as a function of the deformation cycle number. (**f**) Amplitude of each fiber family identified by the FINE algorithm in the anisotropic sample as a function of the deformation cycle number.

To analyze the micro-structural differences between both samples in detail, we track the amplitudes and dispersions of fiber families which are identified by the FINE algorithm. These are shown for both samples in Fig. 4.

The measured force of the isotropic sample is shown in Fig. 4a. Amplitudes and dispersion parameters of the identified fiber families are plotted in 4b,c, respectively. Throughout the entire deformation protocol, the isotropic sample is characterized by two collagen fiber families. The dispersion parameter of fiber family 1 falls below the threshold of an isotropic distribution except for a few data points located at maximum relaxation of the first deformation cycle. Fiber family 2, however, strongly fluctuates within the domain of a high alignment up to dispersion parameter values of 0.3. In the initial, unstressed state, $70\,\%$ of the fibers are contained in the isotropic fiber family 1.

The corresponding measured force of the anisotropic sample is shown in Fig. 4d. Amplitudes and dispersion parameters of the identified fiber families are plotted in Fig. 4e,f, respectively. The collagen network of the anisotropic sample is characterized by two aligned fiber families. In the initial state before the first stretching cycle both fiber families have equal amplitudes and equal dispersion parameter values of 0.10 and 0.09. Upon stretching, we find a similar behavior for both samples. Fibers migrate from fiber family 1 to fiber family 2 with an periodically oscillating amplitude maximizing to $\sim 60\,\%$. One fiber family is oriented along the force direction as indicated by the OI of Fig. 3a,c increasing upon stretching. Furthermore, the dispersion parameter of fiber family 2 of the isotropic sample increases continuously in the relaxed state being responsible for its permanently increased AI.

### Morphological changes and mechanical behavior upon stretching

**Figure 5:**
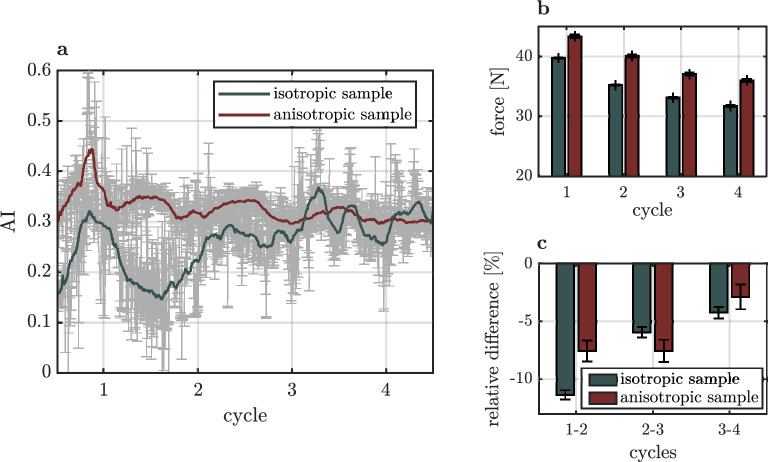
Relation between measured force and orientation of the collagen fiber network. (**a**) Alignment index of the isotropic sample and the anisotropic sample as a function of the deformation cycle number. (**b**) Relative maximum force applied to the isotropic and the anisotropic sample as a function of the deformation cycle number. (**c**) Relative difference of the maximum force of each deformation cycle of the isotropic and the anisotropic sample.

The relation between the AI of both skin samples and their respective mechanical behaviors is shown in Fig. 5. The AI of both samples is shown in Fig. 5a. As pointed out before, the AI of the anisotropic sample constantly fluctuates around a value of 0.3, while the collagen network of the isotropic sample approaches this value after two deformation cycles. As shown in Fig. 5b and in Supplementary Fig. 4, the force at maximum stretches decreases for samples with each deformation cycle, which is in line with stress strain curves measured in literature^7,8,18^. This altered mechanical response of soft tissue due to repeated deformation cycles is referred to as preconditioning effect^24^. In capsular ligaments Quinn et al.^25^ correlated this effect with a permanent alignment of collagen fibers along the direction of force. Here, we note that for both samples, the degree of maximum orientation of the collagen network is only determined by the maximum stretch, since the maximum of the OI of the first deformation cycle is not exceeded by performing additional deformation cycles. A permanent increase of the OI and the AI of collagen fibers in the relaxed state can be observed in case of the isotropic sample. The alignment of the collagen networks of both samples becomes identical after two deformation cycles. This is microscopically reflected by the relative change of the maximum force between each deformation cycle, shown in Fig. 5c. The large relative difference of $-12\,\%$ between maximum forces of the first two cycles of the isotropic sample correlates with the permanent alignment of fibers. Once fibers align to an AI of 0.3, the relative difference of maximum forces between consecutive deformation cycles becomes identical for both samples.

In this proof-of-principle study we introduced an approach to perform mechanical deformations of biological samples while analyzing its collagen microstructure using SHG imaging and the FINE algorithm. The initial results suggest that repeated stretching and relaxation of skin is found to decrease the ability of the collagen fiber network to align in the direction of force indicating a plastic material behavior. Plasticity might be induced by repeatedly stretching the skin above its physiological limit in the dorsal region affecting collagen cross-links. Collagen cross-links are reported to determine the strength of the collagen fiber network at quasi-static strain rates^26^. Nevertheless, in order to prove the biological findings of this proof-of-principle study, further samples need to be analyzed. Additional information on the mechanical behavior of dorsal pig skin can be found in the literature^27,28^. It should be noted that different liquids, namely water, saline or phosphate-buffered saline solutions (PBS) are used for moisturizing biological samples during mechanical deformation^8,27,29^.

This setup aims to study skin deformation using quasi-static strain rates in order to catch slow effects such as aging, long-term loads. At higher strain rates skin exhibits a stiff material response^18,30^. This is related to additional effects like the interaction of collagen fibers with the ground substance as well as the relative sliding of collagen fibers against each other^9,26^. Please note that the bio-mechanical findings of this work are found in response to supra-physiological strains. Future experiments using a maximum strain within the physiological range of dorsal skin will clarify if these findings are maintained in an in-vivo scenario.

In conclusion, we are able to resolve different micro-structural variations of the collagen fiber network of skin, that are related to the interplay of its fiber families. Permanent as well as periodic processes of the collagen fiber network due to cyclic deformations are identified. Furthermore, differences across the samples in terms of their mechanical response are successfully correlated with their individual collagen fiber networks and thus to skin morphologies. Our proof-of-principle study opens a new avenue to investigate biological fiber-reinforced tissue upon mechanical strain. The FINE algorithm in combination with the multiphoton stretching device represents a sophisticated method to relate micro-structural properties to the macroscopic mechanical behavior.

## Methods

### Sample preparation

A back skin of a $\sim 90$ days-old female pig (*Viktoria* breed) was acquired from a local, commercial butcher. Rectangular skin samples were punched with an orientation parallel to the spine and stored in the freezer at $-24^\circ$ until usage. The location of the samples is illustrated in Supplementary Fig. 2. Directly before the experiment, the subcutaneous fat was cautiously removed using a surgical blade. The skin samples were still frozen to avoid any pre-stress of the collagen fibers. Additionally, samples were cut to dimensions of $(80 \times 6)\,\text {mm}^2$. The thickness of the samples was $(3.0\pm 0.5)\,\text {mm}$. Thawed samples were placed between the two clamps with the dermis pointing towards the multiphoton microscope to image deep dermal layers. Constant hydration of the skin during the experiment was ensured by constantly moistening the samples with room temperature water.

### Multiphoton microscope stretching device

#### Multiphoton microscopy

For second harmonic generation (SHG) measurements we used a multiphoton microscope (DermaInspect) which was developed in collaboration with Jenlab GmbH (Jena, Germany)^31^. To measure the collagen-specific second-harmonic generation (SHG) signal, a femtosecond titanium:sapphire laser (*Mai Tai*, Spectra-Physics, California, USA) at a wavelength of $820\, \text {nm}$ was used together with a $(410\pm 10)\, \text {nm}$ band-pass filter (*AQ 410/20m-2P*, Chroma Technology Corp., Bellows Falls, VT). A water-immersion objective with a 20*x* magnification (*XLUMPlanFl 20x/0.95*, Olympus) captured a $(440 \times 440)\, \mu \text {m}$ field of view with a resolution of $(512\times 512)\,\text {pixels}$. Images were cropped by $100\,\text {pixels}$ since the left border was found to suffer from motion artefacts induced by the scanning mirrors of the microscope.

#### Stretching device

As shown in Fig. 1a, samples were clamped into a custom made traction device, which consists of two opposing linear guide units (*RK Compact 30*, RK Rose+Krieger GmbH, Minden, Germany) with equipped stepper motors and $45\,\text {N}$ load cells (*SML-45*, Interfaceforce e.K., Germany). The stepper motors (*ST4209S1006-B*, Nanotec Electronic GmbH & Co. KG, Feldkirchen, Germany) with encoder (*WEDS5541-A14*, Nanotec Electronic GmbH & Co. KG, Feldkirchen, Germany) allow for a minimum step size of $1\, \mu \text {m}$. The whole set-up was mounted onto a lifting stage to allow for a precise placement under the object lens of the microscope. To minimize displacements of the imaging plane, the skin samples were constrained in both directions along the optical axis. The minimum distance to the objective was ensured by a 3D-printed cylindrical spacer (*Form 2, standard black resin*, Formlabs, Somerville, USA) that was mounted to the microscope. The spacer further guaranteed a constant immersion of the objective, since a certain level of water between objective, spacer and the sample was kept. To avoid a potential sagging of the sample, a 3D-printed supporter was attached to the mounting plate underneath the sample. The stretching device was checked for sample slippage in preliminary tests.

#### Tensile tests with simultaneous imaging

We performed tensile, strain-controlled, mechanical tests in cranial-caudal direction with four consecutive cycles of repeated stretch and relaxation. For a simultaneous deformation and imaging of the sample, tensile tests were performed at low strain rate of $0.075\,\%\!/\!{\text {s}}$. Images were recorded with a mean scan-time of $(2.63\pm 0.06)\, \text {s}$ every $3\,\text {s}$ to ensure reliable sequences of consecutive image acquisition and data storage. Prior to deformation, samples were slightly pre-stretched to a force of $0.2\,\text {N}$ to ensure a uniform starting point. Although stretching and relaxation was performed in opposing directions along the specimen’s long axis, a shift of the field of view in the tensile direction could ultimately not be prevented. We took advantage of the piezo element to adjust residual displacements in the direction of the optical axis. Distorted images were omitted from quantitative analysis. However, a sufficient number of images was captured at every point of the deformation curve ensuring a continuous tracking of the dermal collagen fiber network throughout the entire measurement.

### Image processing

#### Angular orientation distribution

We used a Fourier-based method^20^ to obtain the angular orientation distribution $I(\theta )$ of the unprocessed SHG images. Fourier-based methods make use of the power spectrum, defined as the absolute square of the Fourier transform of the image, to calculate $I(\theta )$ by means of a radial sum. Within the method, Poissonian photonic noise of the measured SHG image is assumed^20^. Measurement uncertainties are then propagated to the Fourier domain. These uncertainties are used to define a filter by means of a relative error constrain on the power spectrum. In addition, the uncertainty of the angular orientation spectrum, $\Delta I(\theta )$ is achieved. $I(\theta )$ is used for the computation of the orientation index (OI) (equation 2). Since the FINE algorithm^21^ is based on the cumulative orientation distribution $C(\theta )$ and its uncertainty, $\Delta C(\theta )$, both quantities were calculated and passed to the algorithm.

#### FINE algorithm

The FINE algorithm^21^ determines the number of fiber families *N*, their mean orientations $\theta _i$, amplitudes $a_i$ and dispersion parameters $b_i$. The dispersion parameter *b* quantifies the spread of angles around their mean orientation. It can be understood as reciprocal standard deviation, meaning that a large value of *b* indicates a small spread of angles, i.e. an aligned fiber family, and vice versa. Within the FINE algorithm, a single fiber family is modeled with a sigmoid function that respects the semi-circularity of the angular orientation distribution. Fiber families are iteratively added until the deviation between cumulative orientation distribution, $C(\theta )$, and the fitted model is smaller than $3\sigma =3\Delta C(\theta )$^21^.

#### Local fiber orientation

The local fiber orientation was calculated as described previously^21^. First, the local angular orientation spectra of an image $I_p(x,y)$ were calculated, denoted as $I_p(x,y,\theta )$. Local orientation spectra were achieved from applying the inverse Fourier transformation to the wedge-filtered Fourier transform of the image. The main local orientation in each pixel indicates the angle at which the local orientation spectrum reaches its maximum intensity. The color-coded local main orientation was scaled by the relative intensity of each pixel. To enhance the contrast of fibers, the background signal was removed from the image by using the function *Subtract Background* of the open-source platform Fiji^32^ with a rolling ball radius of $50\,\text {pixels}$.

## Supplementary Information

Supplementary Information.
