# Supplementary material for: A method to analyze the influence of mechanical strain on dermal collagen morphologies
Source: Sci Rep. 2021 Apr 7;11:7565. doi: 10.1038/s41598-021-86907-7 (PMC8027212; doi:10.1038/s41598-021-86907-7)
Supplement: Supplementary file 1 — Supplementary Information. [file 41598_2021_86907_MOESM1_ESM.docx]

**Supplementary information**

**A method to analyze the influence of mechanical strain on dermal collagen morphologies**

Maximilian Witte^1,2^, Michael Rübhausen^1^, Sören Jaspers^2^, Horst Wenck^2^, and Frank Fischer^2 ,^*

1. Center for Free-Electron Laser Science (CFEL), University of Hamburg, Hamburg, 22607, Germany
2. Beiersdorf AG, Hamburg, 20245, Germany

- [Frank.Fischer@beiersdorf.com](mailto:Frank.Fischer@beiersdorf.com)

**Supplementary Figures**

| 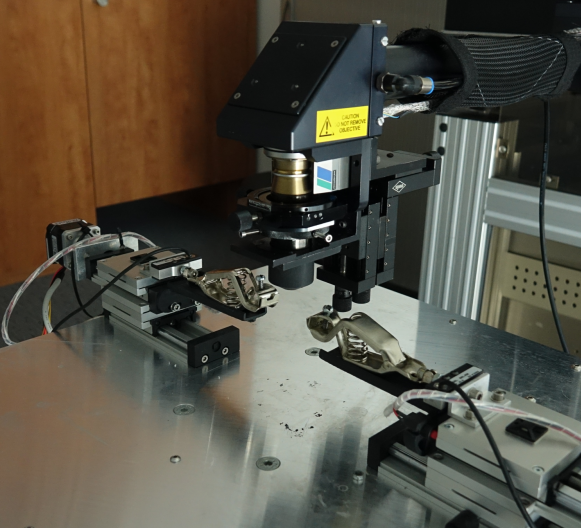 |
| --- |
| **Supplementary Figure 1.** **Multiphoton microscope stretching device**. Skin samples are clamped between two linear rigs that are controlled by stepper motors. Each clamp is attached to a force sensor. The entire stretching unit is mounted onto a lifting column. The objective of the multiphoton microscope is covered by a 3D-printed spacer, which ensures a minimum distance between objective and sample and a constant immersion of the objective. Note that the supporter, which prevents sample sagging during measurement, is not shown. |
| \| 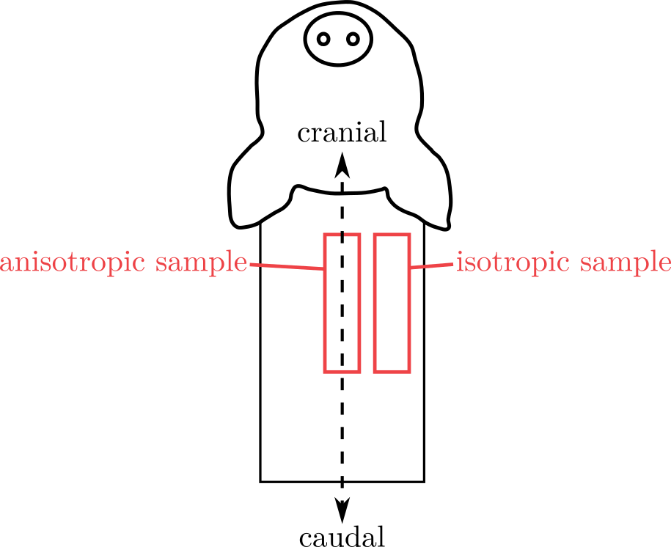 \| \| --- \| \| **Supplementary Figure 2.** **Original locations of the anisotropic and the isotropic sample.** Note that the samples are identified as anisotropic and isotropic depending on their unstressed collagen fiber alignment as measured by SHG imaging. \| |
| \| \| 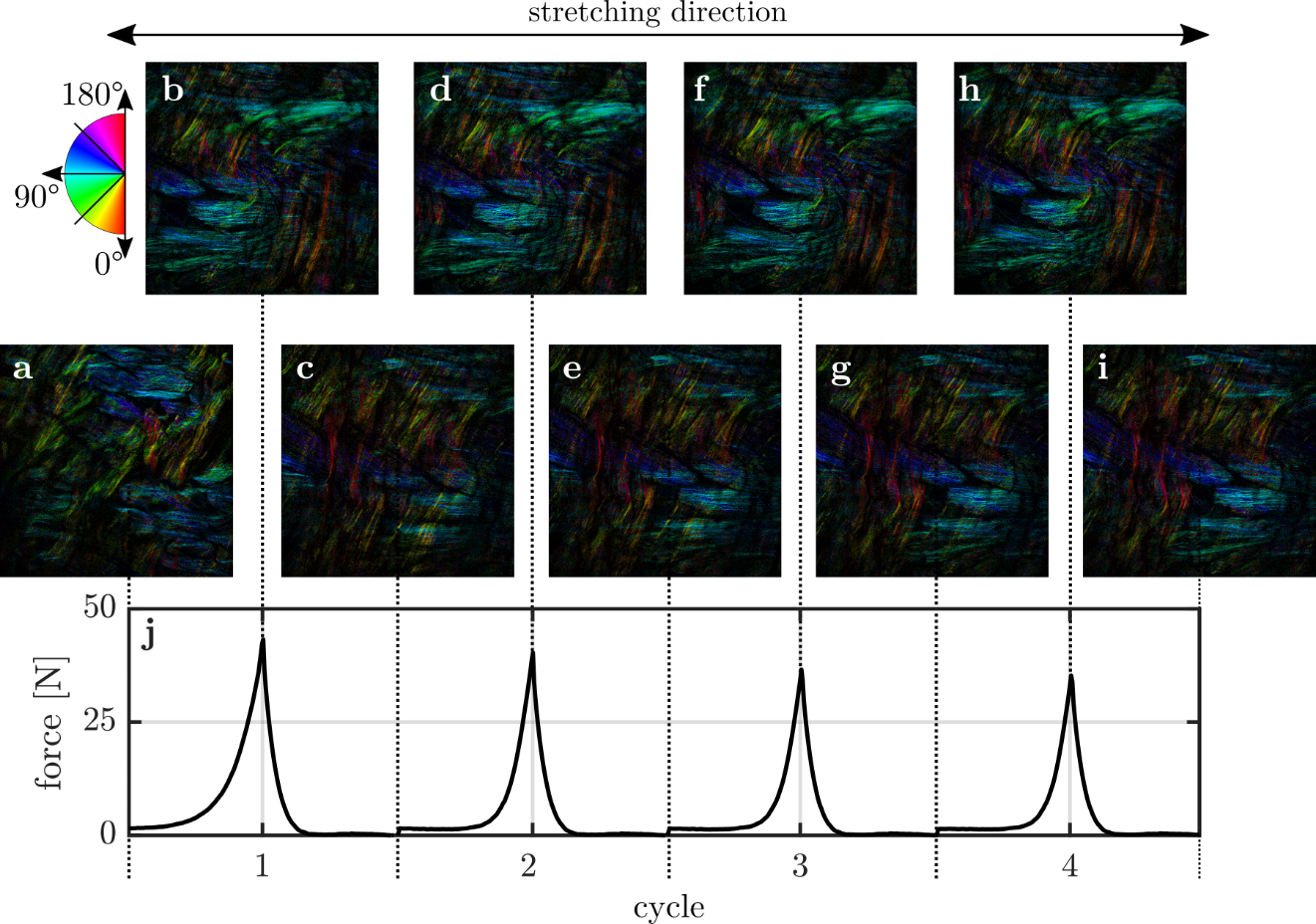 \| \| --- \| \| **Supplementary Figure 3.** **Evolution of the local collagen fiber orientation of the anisotropic skin sample due to repeated stretching and relaxation.** Local fiber orientations are shown in false colors. Dashed lines indicate the minimum and maximum strains of the corresponding stretching cycle. (**a**) Collagen fiber orientation prior to deformation. The sample is stretched in the 90° direction as indicated by the arrow. (**b**) Local fiber orientation at maximum stretch of the first deformation cycle. The corresponding local orientations at maximum stretch of the second, third, and fourth deformation cycle are shown in (**d**), (**f**), and (**h**), respectively. (**c**) Local fiber orientation at maximum relaxation of the first deformation cycle. Similarly, local orientations at minimum stretch of the second, third, and fourth deformation cycle are shown in (**e**), (**g**) and (**i**), respectively. (**j**) Averaged force acting on the sample, measured by the force sensors as a function of the deformation cycle. \|   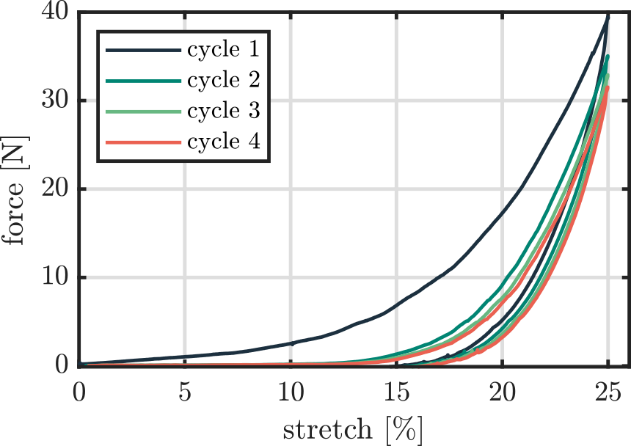 \| \| --- \| --- \| --- \| \| **Supplementary Figure 4.** **Measured force of the isotropic sample as a function of stretch.** A total of four repeated cycles with successive stretching and relaxation are applied to the skin sample displaying the preconditioning effect. \| |
